# BTLA Interaction with HVEM Expressed on CD8^+^ T Cells Promotes Survival and Memory Generation in Response to a Bacterial Infection

**DOI:** 10.1371/journal.pone.0077992

**Published:** 2013-10-30

**Authors:** Marcos W. Steinberg, Yujun Huang, Yiran Wang-Zhu, Carl F. Ware, Hilde Cheroutre, Mitchell Kronenberg

**Affiliations:** 1 Division of Developmental Immunology, La Jolla Institute for Allergy and Immunology, La Jolla, California, United States of America; 2 Infectious and Inflammatory Diseases Center, Sanford|Burnham Medical Research Institute, La Jolla, California, United States of America; MRC National Institute for Medical Research, United Kingdom

## Abstract

The B and T lymphocyte attenuator (BTLA) is an Ig super family member that binds to the herpes virus entry mediator (HVEM), a TNF receptor super family (TNFRSF) member. Engagement of BTLA by HVEM triggers inhibitory signals, although recent evidence indicates that BTLA also may act as an activating ligand for HVEM. In this study, we reveal a novel role for the BTLA-HVEM pathway in promoting the survival of activated CD8^+^ T cells in the response to an oral microbial infection. Our data show that both BTLA- and HVEM-deficient mice infected with *Listeria monocytogenes* had significantly reduced numbers of primary effector and memory CD8^+^ T cells, despite normal proliferation and expansion compared to controls. In addition, blockade of the BTLA-HVEM interaction early in the response led to significantly reduced numbers of antigen-specific CD8^+^ T cells. HVEM expression on the CD8^+^ T cells as well as BTLA expression on a cell type other than CD8^+^ T lymphocytes, was required. Collectively, our data demonstrate that the function of the BTLA-HVEM pathway is not limited to inhibitory signaling in T lymphocytes, and instead, that BTLA can provide crucial, HVEM-dependent signals that promote survival of antigen activated CD8^+^ T cell during bacterial infection.

## Introduction

During acute microbial infections, antigen-specific naïve T cells recognize foreign antigens (Ag), undergo proliferative expansion, differentiate and carry out effector functions. Subsequently, this antigen-specific population undergoes a precipitous decline, but the surviving cells constitute the population of protective memory T lymphocytes. This sequence of events is the hallmark of the adaptive immune response, and the ability to develop and maintain memory T cells is not only a requirement for immune protection against the enormous diversity of pathogens, but it is a goal for effective vaccination against intracellular pathogens.

The development and maintenance of T cell responses that induce optimal protective immunity requires signaling events that involve the interaction between costimulatory or coinhibitory receptors with their ligands [1], [2]. The B and T lymphocyte attenuator (BTLA) is an Ig super family protein with an intermediate type Ig fold in the ectodomain and an ITIM inhibitory signaling domain in the cytosol [3]. BTLA interacts with the herpesvirus entry mediator (HVEM; TNFRSF14), a TNFR super family member. Engagement of BTLA by HVEM, induces tyrosine phosphorylation of the ITIM motifs in the cytoplasmic tail of BTLA, allowing the recruitment of the phosphatases SHP-1 and SHP-2, which attenuate signaling [3], [4]. In addition to the binding to BTLA, HVEM serves as a receptor for four other ligands. It can bind two members of the TNF super family; LIGHT (TNFSF14) and lymphotoxin α (LTα) although its binding with LTα is relatively weak [5]. Furthermore, HVEM can function as the receptor for the herpes simplex virus glycoprotein D (HSV-1 gD), which allows HSV-1 and 2 entry into cells [6], [7]. More recently, CD160 was identified as a second Ig-domain containing molecule able to bind HVEM [8].

Whereas the LIGHT-HVEM interaction participates in T cell costimulation and pro-inflammatory processes, the binding of HVEM with BTLA is in many circumstances anti-inflammatory. For example, HVEM- as well as BTLA-deficient T cells are hyper-responsive to TCR-induced stimulation *in vitro* [3], [4], [9]. In addition, both HVEM and BTLA-deficient mice have a higher susceptibility to experimental autoimmune encephalomyelitis [3], [9], while the loss of BTLA dramatically accelerated partially MHC-mismatched cardiac allograft rejection [10] and prolonged airway inflammation [11]. Similarly, an inhibitory role for BTLA and HVEM has been reported in the Con-A induced hepatitis model [12], and we found previously that the HVEM-BTLA interaction is also critical to prevent severe disease and destructive inflammatory immune responses in a colitis model induced by the transfer of naïve CD4^+^ T cells into *Rag* ^−/−^ mice [13].

Despite exerting an inhibitory role in diverse immune responses, emerging evidence indicates that BTLA can also initiate pro-survival signals for effector T cells. BTLA recently has been reported to play a role in promoting the survival of activated T cells in mouse models of graft versus host disease (GVHD) [14], [15], and effector T cells during development of colitis in the CD4^+^CD45RB^high^ T cell transfer model [13]. Whereas the current understanding of the physiological context of the inhibitory signaling initiated through the HVEM-BTLA pathway is unidirectional, with BTLA functioning as an inhibitory receptor, BTLA can also function as an activating ligand for HVEM promoting NF-κB activation [16], which could promote cell survival. Interestingly, T cells co-express HVEM and BTLA, and HVEM and BTLA interact in either the conventional *trans* configuration, that is between cells, or in the more unconventional *cis* configuration [17]. While in *trans*, bidirectional signaling occurs, with BTLA engaging HVEM to activate NF-κB signaling. In the *cis* configuration, however, BTLA-HVEM binding antagonized NF-κB activation, suggesting that in *cis* these molecules may have a largely inhibitory function [17].

The wide expression of HVEM and BTLA within the immune system, the ability of these two molecules to interact in a *cis* or *trans* configuration, and the capacity of HVEM to bind to multiple ligands, allows for a system of bidirectional signaling interactions with the potential to carry out molecular interactions that have different biological consequences. In this manuscript, we investigated the role of BTLA-HVEM pathway in CD8^+^ T cell immune responses to oral infection caused by the gram-positive intracellular bacterium *Listeria monocytogenes*.

## Results

### Reduced Accumulation of Antigen-specific CD8^+^ T Cells in *Btla* ^−/−^ Mice

**Figure 1:**
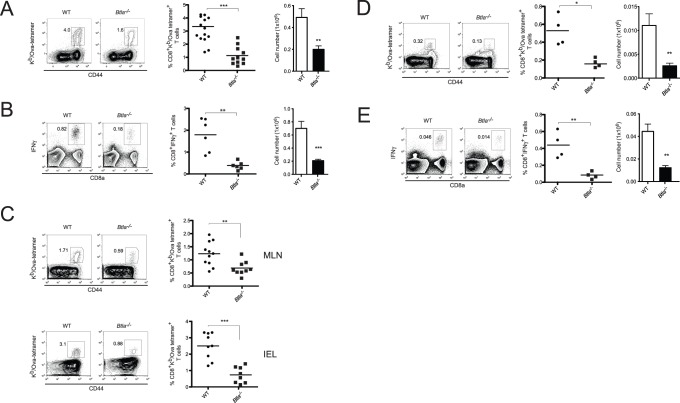
Decreased number of antigen-specific CD8^+^ T cells in *Btla* ^−/−^ mice after LM-OVA infection. A- WT and *Btla* ^−/−^ mice were orally infected with 1×10^9^ LM-OVA and 7 days p.i., OVA-specific CD8^+^ T cells were detected by staining spleen cells with OVA-tetramer (K^b^/OVA_257–264_). The percentage and absolute number of OVA-tetramer^+^ CD8^+^ T cells found in WT (circles and open bars) and *Btla* ^−/−^ (squares and filled bars) mice are shown. Each symbol represents an individual mouse from a total of 11–14 mice per group, pooled from three independent experiments. B- IFNγ production by CD8^+^ T cells isolated from WT and *Btla* ^−/−^ mice, 7 days p.i., was calculated by intracellular staining after *ex vivo* stimulation of the cells with OVA_257–264_ peptide. The percentage and absolute number of IFNγ^+^CD8^+^ T cells is shown. Data shown correspond to the average of 4–6 mice per group and are representative of three independent experiments. C- OVA-tetramer staining in MLN cells and IEL isolated from LM-OVA infected animals, 7 days p.i. Data pooled from three independent experiments are shown. D- Reduced number of memory CD8^+^ T cells in *Btla* ^−/−^ mice. The percentage and absolute number of OVA-specific memory CD8^+^ T cells in the spleen of LM-OVA-infected animals was calculated by staining cells with OVA-tetramers, 70 days p.i. E- IFNγ intracellular staining performed on CD8^+^ T cells isolated from infected mice 70 days p.i., restimulated *ex vivo* with OVA_257–264_ peptide. Data shown correspond to the average of 4 mice per group and are representative of three independent experiments.

In naïve mice, BTLA is expressed by CD8^+^ T lymphocytes [17] (and see **Figure S1**). To investigate the role of BTLA in CD8^+^ T cell responses during an acute bacterial infection, WT or *Btla* ^−/−^ mice were orally infected with recombinant *Listeria monocytogenes*-expressing ovalbumin (LM-OVA). Seven days post-infection (p.i.), at the peak of the immune response, the number of antigen-specific CD8^+^ T cells in the spleen, mesenteric lymph nodes (MLN) and in intraepithelial lymphocytes (IEL) in the small intestine of the infected mice were evaluated. Despite the inhibitory function often attributed to BTLA, we found significantly lower percentages and absolute numbers of antigen-specific CD8^+^ T cells in the spleen of BTLA-deficient mice (**Figure 1A**). Consistently, we also observed lower percentages and numbers of IFNγ-producing CD8^+^ T cells after *ex vivo* re-stimulation of the *Btla* ^−/−^ cells with the SIINFEKL peptide epitope (**Figure 1B**). *Btla* ^−/−^ mice also had two to three times fewer OVA-specific T cells in the MLN and IEL (**Figure 1C**). Moreover, the number of CD8^+^ T cells secreting IFNγ in the MLN of *Btla* ^−/−^ animals following *ex vivo* re-stimulation with OVA peptide was also reduced compare to WT mice (**figure S2**).

To investigate if BTLA deficiency influenced the accumulation of memory CD8^+^ T cells, we measured the number of OVA-specific CD8^+^ memory T cells in WT and *Btla* ^−/−^ mice, 70 days p.i. The percentages and absolute numbers of CD8^+^ tetramer^+^ T cells in the spleen of BTLA-deficient mice were significantly lower than those in WT mice (**Figure 1D**). Consistent results were obtained when cells were re-stimulated *ex vivo* and the number of IFNγ-producing cells was evaluated. The percentage and number of IFNγ^+^CD8^+^ T cells in the spleen of WT animals was approximately three times higher than that in *Btla* ^−/−^ mice (**Figure 1E**). Similar results we obtained when OVA-specific cell numbers were analyzed in the MLN and the intestinal epithelium (**data not shown**). These surprising results demonstrate that BTLA is required for the effective accumulation of primary effector and memory CD8^+^ T cells following LM-OVA infection.

### Expression of BTLA by T Cells is Dispensable for CD8^+^ T Cell Accumulation

**Figure 2:**
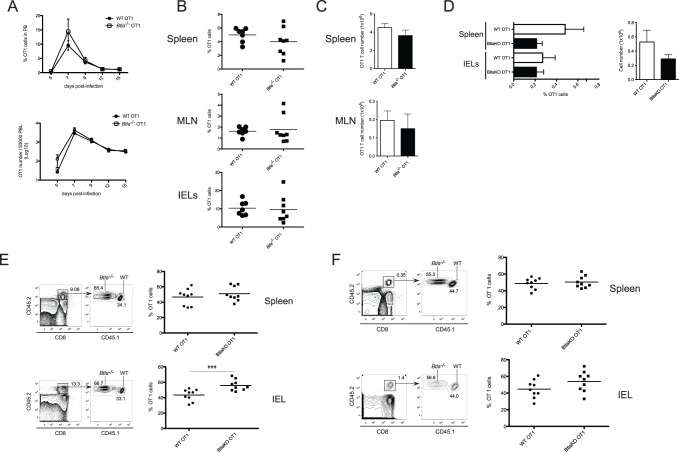
BTLA expression on OT1 cells is not required for their accumulation after LM-OVA infection. Purified naïve WT or *Btla* ^−/−^ OT1 cells were adoptively transferred into CD45.1^+^ recipient mice and one day post transfer, mice were orally infected with LM-OVA as indicated in Material and Methods. A- The percentage (top panel) and absolute number (bottom panel) of donor WT and *Btla* ^−/−^ OT1 cells in the peripheral blood of infected mice was tracked at different time points after infection. OT1 cell numbers in the peripheral blood were calculated as numbers of OT1 cells per 3×10^4^ peripheral blood lymphocytes (PBL). Data shown correspond to the average of 6–8 mice per group. B- Percentages of CD45.2^+^ WT and *Btla* ^−/−^ OT1 cells in the spleen, MLN and IEL of infected mice, 7 days p.i. Each symbol represents an individual mouse from a total of 7–9 mice per group, pooled from two independent experiments. C- Absolute number of WT (open bars) and *Btla* ^−/−^ (filled bars) OT1 cells in the spleen and MLN of infected animals at 7 days p.i. Data shown correspond to the average of the number of animal indicated in B. D- Percentage of OT1 memory cells in the spleen and IEL of WT (open bars) and *Btla* ^−/−^ (filled bars) mice, 70 days p.i. Absolute number of OT1 cells in the spleen of these animals is also represented. Differences between percentage and absolute numbers of WT and *Btla* ^−/−^ OT1 are not statistically significant. Data shown was pooled from two independent experiments with total of 7–9 mice per group, and E, F- Co-transfer experiment with 5×10^4^ CD45.1^+^CD45.2^+^ WT and CD45.2^+^ *Btla* ^−/−^ naïve OT1 cells transferred into CD45.1^+^ recipient mice that were subsequently infected with LM-OVA. Data shown correspond to the percentage of WT (circles) and *Btla* ^−/−^ (squares) cells within the total OT1 cells found in the spleen and IEL of infected mice, 7 days (E) and 70 days (F) p.i. Each symbol represents an individual mouse from a total of 9 mice per group, pooled from two independent experiments.

To investigate if expression of BTLA by CD8^+^ T cells is involved in promoting the accumulation of effector and memory T cells after LM-OVA infection, we crossed *Btla* ^−/−^ mice to OT1 TCR transgenic mice, in which T cells express the OVA_257–264_K^b^-specific TCR. Naïve (CD44^low^) WT or BTLA-deficient CD8^+^ T cells were sorted by flow cytometry and 5×10^4^ purified cells were adoptively transferred into congenic CD45.1^+^ WT recipients. OT1 T cell expansion was measured in peripheral blood isolated at various times after LM-OVA infection. Surprisingly, the expansion of WT and *Btla* ^−/−^ OT1 cells in the peripheral blood of infected hosts was essentially equivalent. Although at early times after infection there was a trend towards a more rapid accumulation of *Btla* ^−/−^ transgenic T cells, this was not statistically significant and comparable OT1 cell numbers were found at the peak of the clonal expansion, seven days p.i., as well as during the contraction phase, between days 9 and 15 (**Figure 2A**). To further analyze the role of BTLA on OT1 cell expansion, we assessed the percentage and number of transferred WT or *Btla* ^−/−^ OT1 cells in the spleen, MLN and IEL of infected recipients. Consistent with the results observed in peripheral blood, the percentages (**Figure 2B**) and absolute numbers (**Figure 2C**) of OT1 cells found in these sites at the peak of the immune response were similar. We also found no significant differences between the number of WT and *Btla* ^−/−^ OT1 cells, 70 days p.i., indicating that BTLA on T cells is dispensable for memory CD8^+^ T cell accumulation (**Figure 2D**). To further investigate the requirement of BTLA during CD8^+^ T cell differentiation in response to LM-OVA infection, we performed competitive adoptive co-transfers with equal numbers of WT (CD45.1^+^CD45.2^+^) and *Btla* ^−/−^ (CD45.2^+^) OT1 cells transferred into CD45.1^+^ hosts. In agreement with the results obtained in the single transfer experiments, WT and BTLA-deficient OT1 cells expanded similarly in the spleen of infected mice (**Figure 2E**). At the peak of the response, however, the percentage of *Btla* ^−/−^ OT1 cells found in the intestine epithelium of these animals was significantly higher than WT cells (**Figure 2E**). Because BTLA can act as an inhibitory receptor, this finding in IEL likely reflects an enhanced proliferative capacity of the cells from the knockout mice during the priming and activation phases of the T cell response. Despite this early proliferative advantage, the absence of BTLA did not greatly influence the generation of memory CD8^+^ T cells, because 70 days after LM-OVA infection the percentages of WT and *Btla* ^−/−^ OT1 found in the spleen and intestine of infected hosts were comparable (**Figure 2F**). These findings indicate that expression of BTLA in CD8^+^ T cells is not absolutely required for the optimal generation of effector and memory CD8^+^ T cells in this experimental system.

### BTLA Expression in the Host is Required for CD8 T Cell Expansion/Survival

**Figure 3:**
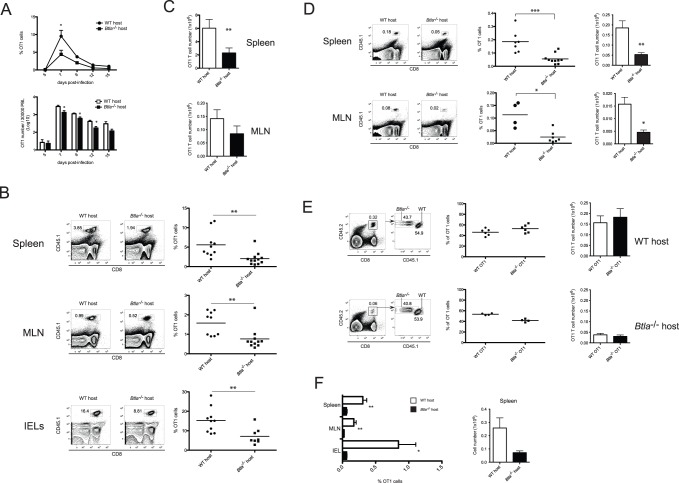
BTLA expression in the host environment is required for expansion/survival of CD8 T cells. 5×10^4^ purified naïve CD45.1^+^ WT CD8^+^ OT-1 cells were adoptively transferred into WT or Btla^−/−^ recipient mice. One day after transfer, mice were orally infected with 1×10^9^ LM-OVA. A- The percentage and absolute number of donor CD45.1^+^ OT1 cells in the peripheral blood of WT (circles and open bars) and *Btla* ^−/−^ (squares and filled bars) hosts was tracked at different time points following infection. OT1 numbers in the peripheral blood were calculated as indicated in figure 2A. Percentages (B) and absolute numbers (C) of CD45.1^+^ OT1 cells in WT and *Btla* ^−/−^ recipient mice, 7 days p.i. Data pooled from three independent experiments is shown. D- Percentage and absolute number of memory OT1 cells in the spleen and MLN of WT and *Btla* ^−/−^ recipient mice, 70 days p.i. Data pooled from two independent experiments is shown. E- Co-transfer experiments with WT (CD45.1^+^CD45.2^+^) and *Btla* ^−/−^ (CD45.2^+^) naïve OT1 cells transferred into congenic CD45.1^+^ WT or *Btla* ^−/−^ recipients. The percentage and absolute number of CD45.2^+^ WT or *Btla* ^−/−^ cells within total OT1 cells found in the spleen of transferred recipients measured 70 days p.i., is shown. F- Percentage (left) and absolute number (right) of total OT1 cells (CD45.2^+^) in the spleen, MLN and IEL of co-transferred WT and BTLA-deficient recipient mice, 70 days after LM-OVA infection.

Whereas many studies have focused on the role of BTLA expressed by T lymphocytes, BTLA expression has been detected on other lymphoid and myeloid cells, and therefore, expression by one or more of these immune cell types could be required for the generation of effector and memory CD8^+^ T cells. To address this possibility, we adoptively transferred 5×10^4^ naïve WT (CD45.1^+^) OT1 cells into CD45.2^+^ WT or *Btla* ^−/−^ hosts that were subsequently infected with LM-OVA, and analyzed for the presence of OT1 cells among peripheral blood lymphocytes (PBL) at several times p.i. The number of OT1 cells in the blood of WT and BTLA-deficient mice was similar at day 5 p.i. (**Figure 3A**). This observation suggests that the absence of BTLA in the host did not impede the proliferation and expansion of WT OT1 cells early following LM-OVA infection. However, at the peak of the immune response, the number of OT1 cells was significantly lower in BTLA-deficient hosts. Indeed, these mice had a marked reduction in the percentage and absolute number of Ag specific T lymphocytes (**Figure 3A**). This difference was maintained during the contraction phase of the response, indicating that the reduced accumulation of OT1 cells in *Btla* ^−/−^ mice was not primarily due to a delayed accumulation/expansion of the cells (**Figure 3A**). We further characterized OT1 cell-accumulation by measuring the number of OVA-specific cells in different sites from WT and BTLA knockout mice after infection. Similar to the results in peripheral blood, the percentages of OT1 cells found in *Btla* ^−/−^ animals were significantly lower than those in WT hosts, with an approximately 2-fold decrease in the percentages of OT1 cells in the spleen, MLN and the small intestine of BTLA-deficient mice (**Figure 3B**). Moreover, the absolute number of the OT1 cells was also considerably lower in these hosts (**Figure 3C**).

In accord with the results presented earlier showing a reduced endogenous response and generation of memory CD8^+^ T cells in *Btla* ^−/−^ mice (Figure 1D,E), we observed considerably lower numbers of memory OT1 cells in the spleen, MLN and the small intestine (**Figure 3D** and data not shown) of BTLA-deficient hosts, 70 days p.i. These data indicate that BTLA expression in the host is necessary for the accumulation of both primary effector and memory CD8^+^ OT1 T cells.

When WT cells are transferred into a gene deficient host, the host immune system might reject the WT cells. This is an important issue, because the reduced numbers of OT1 cells observed in *Btla* ^−/−^ mice could be caused by rejection of the WT (*Btla* ^+/+^) cells transferred into a BTLA-deficient environment. To address this issue, we adoptively co-transferred equal numbers of WT and *Btla* ^−/−^ OT1 cells into CD45.1^+^WT or CD45.1^+^BTLA-deficient recipients that were then infected with LM-OVA. The rationale of this experiment was that if an immunological rejection of BTLA^+^ cells happened, then the number of WT OT1 cells in *Btla* ^−/−^ hosts should be lower compared to OT1 cells lacking BTLA. In WT hosts, rejection should not occur and therefore the ratio between both OT1 cell subsets should be maintained. When the percentages and numbers of WT and *Btla* ^−/−^ OT1 cells were measured 70 days p.i., we found an approximately 1∶1 ratio in all infected hosts, independently of their *Btla* genotype (**Figure 3E**). When we compared the amount of total (*Btla* ^+/+^+*Btla* ^−/−^) OT1 cells recovered from WT and *Btla* ^−/−^ infected mice, we observed reduced percentages and numbers of transgenic T cells in the recipients lacking BTLA (**Figure 3F**). These results indicate that BTLA expression in the host is critical for the accumulation of both WT and *Btla* ^−/−^ effector T cells, and the generation of normal numbers of memory CD8^+^ T lymphocytes after LM-OVA infection.

### Reduced Accumulation of Antigen-specific CD8^+^ T Cells in *Hvem* ^−/−^ Mice

**Figure 4:**
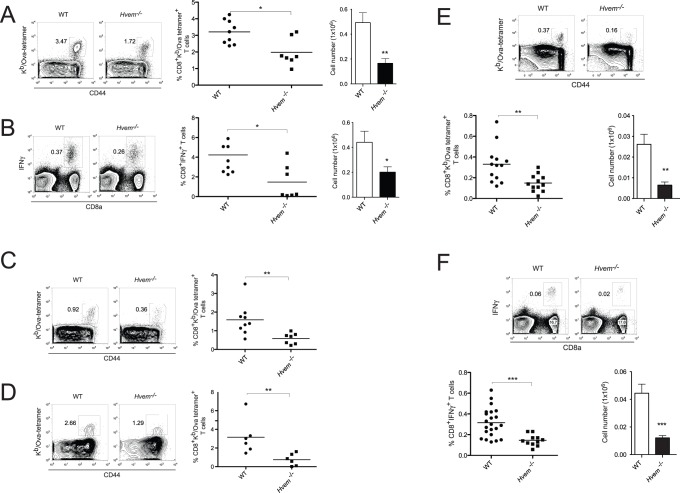
Reduced accumulation of antigen-specific CD8^+^ T cells in *Hvem* ^−/−^ mice. WT or *Hvem* ^−/−^ mice were orally infected with 1×10^9^ LM-OVA and the amount of antigen-specific CD8^+^ T cells was measured. A- Percentage and absolute number of OVA-specific CD8^+^ T cells in splenocytes isolated from WT or *Hvem* ^−/−^ mice, 7 days p.i. B- IFNγ production by WT or *Hvem* ^−/−^ splenic CD8^+^ T cells after *ex vivo* restimulation with OVA_257–264_ peptide. The percentage of OVA-specific CD8^+^ T cells in the MLN (C) and IEL (D) of WT and *Hvem* ^−/−^ mice was also calculated, 7 days after infection. Data shown correspond to 6–9 mice per group, pooled from two independent experiments. E- Percentage and absolute number of memory OVA-specific CD8^+^ T cells in the spleen of WT and *Hvem* ^−/−^ mice, 70 days p.i. F- IFNγ production by OVA-specific memory CD8^+^ T cells isolated from the spleen of WT and *Hvem* ^−/−^ mice 70 days p.i. and restimulated *ex vivo* with OVA-peptide. Data were pooled from at least two independent experiments, each one performed with 5–7 mice per group.

We have previously shown that engagement of HVEM by a soluble BTLA:Fc chimeric protein leads to enhanced survival of CD8^+^ T cells stimulated *in vitro* with an anti-CD3ε mAb [16]. This is consistent with other data we have obtained indicating that binding in *trans* of HVEM by BTLA triggers the activation of the NF-κB signaling pathway and pro-survival signals. Most CD8^+^ T cells express HVEM, with memory cells expressing only slightly lower levels than naïve cells (**Figure S1**). Based on these data, we hypothesized that during *Listeria* infection, BTLA expression in the host might promote the survival of effector CD8^+^ T lymphocytes by interacting with HVEM expressed by T cells. To evaluate this possibility, we analyzed effector CD8^+^ T cell differentiation in HVEM-deficient mice infected with LM-OVA. Similar to *Btla* ^−/−^ mice, at the peak of the immune response, HVEM-deficient animals had a reduction in the percentage and number of OVA-specific CD8^+^ T cells in the spleen (**Figure 4A**). Following *ex vivo* re-stimulation with SIINFEKL peptide, the percentages and absolute numbers of IFNγ^+^CD8^+^ T cells in *Hvem^−/−^* mice were also decreased compared with WT mice (**Figure 4B**). The amount of antigen-specific CD8^+^ T cells in MLN (**Figure 4C**) and small intestine epithelium (**Figure 4D**) of the infected mice was also significantly lower in *Hvem* ^−/−^ mice. When we analyzed the generation of memory CD8^+^ T cells in WT and HVEM-deficient animals at 70 days post-infection, the differences observed earlier were maintained, with approximately 2-fold less antigen-specific CD8^+^ T cells in the absence of HVEM (**Figure 4E**). Moreover, HVEM-deficient mice had reduced numbers of IFNγ-producing cells in the memory phase after *ex vivo* re-stimulation with OVA-peptide (**Figure 4F**). These data demonstrate that similar to BTLA-deficiency, the absence of HVEM leads to a reduction in memory CD8^+^ T cells following LM-OVA infection. The common phenotype of a reduction in antigen-specific CD8^+^ T cells in BTLA- and HVEM-deficient mice suggests that the BTLA-HVEM pathway plays a role in the survival of antigen-specific CD8^+^ T cells after *Listeria* infection.

### Expression of HVEM by T Cells Contributes to their Accumulation after Infection

**Figure 5:**
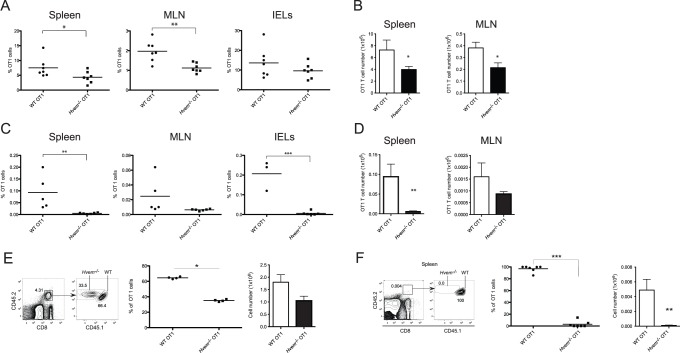
Hvem expression in OT1 cells is required for their accumulation after LM-OVA infection. 5×10^4^ purified naïve WT or *Hvem* ^−/−^ OT1 cells were adoptively transferred into CD45.1^+^ recipient mice. One day after the transfer, mice were orally infected with 1×10^9^ LM-OVA and the amount of transferred OT1 cells was monitored at 7 or 70 days p.i. The percentage of CD45.2^+^ WT and *Hvem* ^−/−^ effector OT1 cells in the spleen, MLN and IEL of the recipient animals (A), as well as the absolute number of these cells in the spleen and MLN of infected mice (B), were measured 7 days p.i. Data shown correspond to 7 mice per group, pooled from two independent experiments. The percentage (C) and absolute number (D) of WT and *Hvem* ^−/−^ memory OT1 cells in the recipient mice were also calculated, 70 days after LM-OVA infection. Data shown are representative of two independent experiments. E-F- Co transfer experiments with equal numbers of naïve WT (CD45.1^+^CD45.2^+^) and *Hvem* ^−/−^ (CD45.2^+^) OT1 cells injected into CD45.1^+^ recipient mice. One day after co-transfer, mice were orally infected with LM-OVA and the percentage of WT and *Hvem* ^−/−^ cells gated on total OT1 cells, as well as the absolute number of both OT1 cell populations was calculated at 7 days (E) and 70 days (F) following infection. Data shown are representative of at least two independent experiments.

To investigate if the expression of HVEM by CD8^+^ T lymphocytes is required for the accumulation of the effector and memory CD8^+^ T cells during *Listeria* infection, we crossed *Hvem* ^−/−^ mice with OVA-transgenic OT1 mice. 5×10^4^ purified naïve WT or *Hvem* ^−/−^ OT1 cells were adoptively transferred into CD45.1^+^ congenic hosts that were then infected orally with LM-OVA. 7 days after LM-OVA infection, the amount of transferred OT1 cells present in the infected hosts was tracked as CD8^+^CD45.2^+^ T cells. Interestingly, the absence of HVEM on OT1 cells led to a reduction in the percentages of these cells in the spleen, MLN and IEL of infected mice, although in the small intestine the differences did not reach statistical significance (**Figure 5A**). Additionally, the absolute number of OT1 cells found in the spleen and MLN of the infected hosts was also significantly lower for cells lacking HVEM (**Figure 5B**). The effect of HVEM-deficiency on OT1 cells became even more evident at the memory phase of the immune response. At day 70 after LM-OVA infection, the percentages and numbers of *Hvem* ^−/−^ OT1 cells found in the spleen, MLN and the small intestine of infected mice were dramatically reduced as compared to WT OT1 cells (**Figure 5C,D**). To further assess the role of HVEM on CD8^+^ T cells, we performed adoptive co-transfer experiments in which equal numbers of WT (CD45.1^+^CD45.2^+^) and *Hvem* ^−/−^ (CD45.2^+^) OT1 cells were transferred into the same hosts (CD45.1^+^) that were then infected with LM-OVA. In this competitive situation, HVEM-deficient OT1 cells also accumulated less than WT cells and at the peak of the response, only a third of the total CD8^+^CD45.2^+^ OT1 cells found in the spleen of infected hosts were from HVEM-knockout donors (**Figure 5E**). Similar to the single transfers, HVEM-deficiency in OT1 cells led to an even more dramatic reduction in the percentages and numbers of memory OT1 cells in the spleen (**Figure 5F**), MLN and IEL (**data not shown**) of infected mice, 70 days p.i. These results demonstrate that expression of HVEM on CD8^+^ T cells is necessary for the optimal accumulation of effector CD8^+^ T cells and for the generation and/or maintenance of the CD8^+^ T cell memory.

### HVEM Expression in CD8^+^ T Cells Promotes Survival

**Figure 6:**
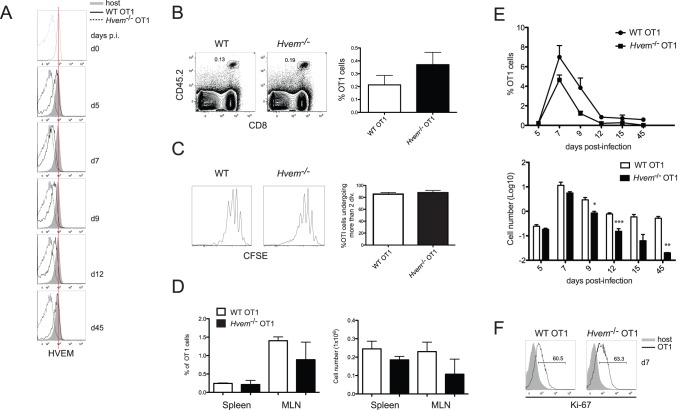
HVEM promotes the survival of OT1 cells after LM-OVA infection. Purified naïve WT or *Hvem* ^−/−^ CD8^+^ OT1 cells were adoptively transferred into CD45.1^+^ recipient mice and one day later, mice were orally infected with LM-OVA. A- HVEM expression on naïve OT1 cells and OT1 cells isolated from peripheral blood of LM-OVA infected mice was assessed at the indicated times after infection. To analyze the early expansion of WT and *Hvem* ^−/−^ CD8^+^ T cells in mice infected with LM-OVA, 5×10^5^ CFSE-labeled WT or *Hvem* ^−/−^ OT1 cells were transferred into CD45.1^+^ recipients and the percentage of OVA-specific T cells in the MLN of the hosts, 4 days p.i., was analyzed (B). Proliferation of WT and *Hvem* ^−/−^ OT1 cells in the MLN of infected mice was assessed by CFSE dilution profiles (C). Histogram shows the percentage of WT (open bar) and *Hvem* ^−/−^ (filled bar) OT1 cells undergoing more than two cell divisions. The accumulation of activated CD8^+^ effector T cells was also monitored at day 5 p.i., by measuring the percentage and absolute number of WT and *Hvem* ^−/−^ OT1 cells in the spleen and MLN of LM-OVA-infected animals (D). The ability of WT and *Hvem* ^−/−^ OT1 cells to expand and contract following LM-OVA infection was determined by tracking the percentage and absolute number of the CD45.2^+^ OT1 cells in the spleen of infected mice at different time points after infection (E). The proliferation of WT and *Hvem* ^−/−^ OT1 cells in the spleen of infected mice at the peak of the immune response was evaluated by measuring the intracellular levels of the proliferation marker Ki-67 (F).

Because *Hvem* ^−/−^ OT1 cell numbers in mice infected with LM-OVA were substantially lower at the peak of the T cell clonal expansion, it appeared that the critical role for HVEM in regulating CD8^+^ T cell responses takes place early after bacterial infection. Therefore, we decided to investigate how HVEM expression on CD8^+^ T cells regulates cell proliferation and survival during the expansion and contraction phases of the immune response elicited by *Listeria* infection. To this end, we transferred equal numbers of WT or *Hvem* ^−/−^ OT1 cells and tracked the transferred cells following infection. We first assessed how HVEM expression on OT1 cells is regulated during an acute bacterial infection. Naïve OT1 cells all expressed surface HVEM before transfer, similar to the endogenous, polyclonal CD8^+^ T cells (**Figure 6A**). Following LM-OVA infection, HVEM expression was slightly down-modulated by day 5 p.i., with a more rapid down-regulation occurring later between days 5 and 7 p.i., and at the peak of the immune response, HVEM expression on OT1 cells was almost undetectable (**Figure 6A**). At the contraction phase, however, HVEM began to be re-expressed on the surface of the OT1 cells and its expression increased from day 9 to 12. Memory-differentiated OT1 cells found 45 days after LM-OVA infection fully regained surface HVEM expression and they had equivalent amounts of HVEM compared to naïve OT1 cells (**Figure 6A**).

HVEM has been previously described as a T cell costimulatory molecule, and it is reasonable to assume that its expression on CD8^+^ T cell is required during priming for optimal activation and proliferation of the antigen-specific T cells. To evaluate this possibility, we adoptively transferred CFSE-labeled naïve WT or *Hvem* ^−/−^ OT1 cells and infected the recipients with LM-OVA. Mice were sacrificed 4 days p.i. and proliferation of the OT1 cells isolated from various tissues was measured based on CFSE-dilution profiles. At this early time point after oral infection, abundant numbers of transferred OT1 cells are usually found in the MLN, where they proliferate and expand in response to oral antigens. When we measured the number of OT1 cells in the MLN of the hosts, we found equivalent percentages of OT1 cells, regardless of the HVEM-genotype (**Figure 6B**). Although a trend towards higher absolute numbers of HVEM-deficient OT1 cells was observed, the differences did not reach statistical significance. When cell proliferation was evaluated, both WT and *Hvem* ^−/−^ OT1 cells were able to proliferate to a similar extent, with 85.5% and 87.8% of the cells, respectively, undergoing between 2 to 5 rounds of cell division (**Figure 6C**). Similar results were obtained when cell numbers and proliferation were measured in cells from the spleens of infected mice (**data not shown**). In agreement with the CFSE dilution results, at day 5 p.i., we detected equivalent percentages and absolute numbers of WT and *Hvem* ^−/−^ OT1 cells in the spleen and MLN of infected mice (**Figure 6D**), indicating a normal early proliferative response in *Hvem^−/−^* mice. However, HVEM-deficient effector T cells failed to accumulate at the same rate as their WT counterparts (**Figure 6E**). The differences became apparent between days 6 and 7 p.i., and at the peak of the effector response, we observed an approximately 1.5-fold decrease in the number of *Hvem* ^−/−^ OT1 cells compare to WT cells (**Figure 6E**). At this time, however, both OT1 cell populations had comparable levels of the proliferation marker Ki-67 (**Figure 6F**), indicating that the difference in the accumulation of the WT and *Hvem* ^−/−^ OT1 cells was not primarily related to a defective proliferative capacity of the HVEM deficient T cells. Importantly, the difference between WT and *Hvem* ^−/−^ OT1 cell numbers became more pronounced during the contraction phase, between days 7 and 15 p.i., when most of the effector CD8^+^ T cells are undergoing apoptosis (**Figure 6E**). As shown earlier, HVEM expression during this phase is progressively regained, consistent with the hypothesis that HVEM expression during the contraction of the antigen-specific CD8^+^ T cell response may protect differentiated effectors from apoptosis. When the expression of KLRG-1, CD127, CD62L, CD122 and CD43 was assessed on WT and *Hvem* ^−/−^ OT1 cells at different times during the immune response, we found equivalent percentages of both OT1 cell populations expressing these markers (**Figure S3**). This data indicates that HVEM deficiency does not affect effector T cell differentiation program but rather the accumulation of both short lived and memory precursor effector cells.

Taken together, our results indicate that HVEM expression contributes to the accumulation of effector and memory CD8^+^ T cells, likely by promoting the survival of activated, antigen-specific CD8^+^ T cells during the latter part of the clonal expansion and the contraction phases of the immune response to LM-OVA.

### BTLA Promotes the Survival of Antigen-specific HVEM-expressing CD8^+^ T Cells

**Figure 7:**
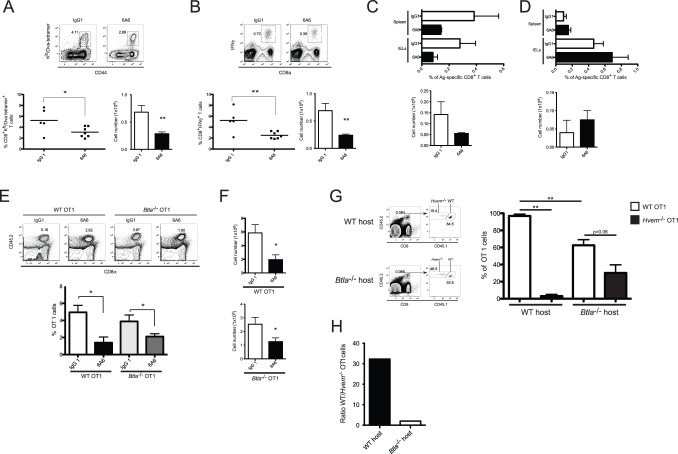
BTLA promotes the long-term survival of activated CD8^+^ T cells through its interaction with HVEM on T cells. WT mice were injected intraperitoneally with an anti-BTLA blocking antibody (clone 6A6) or isotype control (IgG1), one day before and 2 days after oral infection with 1×10^9^ LM-OVA. A,B- 7 days p.i., endogenous OVA-specific CD8^+^ effector T cell response was assessed by OVA-tetramer staining (A) and IFNγ intracellular staining after *ex vivo* stimulation with OVA_257–264_ peptide (B). C- The percentage and absolute number of endogenous OVA-specific memory CD8^+^ T cells in the spleen of mice treated with the anti-BTLA mAb as indicated above, were monitored 70 days post LM-OVA infection. Data shown are representative of two independent experiments. D- To assess the effect of blocking BTLA-HVEM interaction at the memory phase of the immune response, WT mice were orally infected with LM-OVA were treated with the anti-BTLA mAb at days 20, 27, 30 and 34 p.i. Endogenous OVA-specific CD8^+^ memory T cells were measured in the spleen and IEL of the animals, 40 days p.i. Data shown correspond to the average of 8 mice per group pooled from two independent experiments. E,F- To rule out that the anti-BTLA antibody works by engaging BTLA expressed on T cells, WT or *Btla* ^−/−^ OT1 cells were adoptively transferred into CD45.1^+^ WT mice that were treated with the anti-BTLA mAb as described in A. 7 days after LM-OVA infection, the percentage (E) and absolute number (F) of CD45.2^+^ OT1 cells in the recipients were measured. Data shown was pooled from two independent experiments. G,H- Co-transfer experiment with equal numbers of CD45.1^+^CD45.2^+^ WT and CD45.2^+^ *Hvem* ^−/−^ OT1 cells adoptively transferred into CD45.1^+^ WT or CD45.1^+^ *Btla* ^−/−^ recipient mice. The percentage of memory WT and *Hvem* ^−/−^ cells within total OT1 cells in the spleen of the recipients was measured 70 days p.i. (G). The ratio of WT vs. *Hvem* ^−/−^ memory OT1 cells in the different recipients was calculated (H). Data shown correspond to 7 mice per group, pooled from two independent experiments.

Our data indicate that both BTLA and HVEM are required for the accumulation of antigen-specific T cells during LM-OVA infection. To confirm this by pharmacologic inhibition, and to rule out a developmental defect due to germ line gene deletion, we performed experiments using an anti-BTLA (clone 6A6) monoclonal antibody (mAb) that competitively inhibits HVEM-BTLA binding interactions [10], [14], [18]. WT mice were treated with the 6A6 antibody one day prior to LM-OVA infection and again at day 2 p.i. Blockade of the HVEM-BTLA interaction with the anti-BTLA mAb led to a significant reduction in the percentages and absolute numbers of antigen-specific CD8^+^ T cells in the spleen of infected mice at day 7 (**Figure 7A**). Furthermore, after *ex vivo* restimulation with SIINFEKL peptide, decreased numbers of effector CD8^+^ T cells secreting IFNγ were found in cells from mAb treated mice (**Figure 7B**). These results indicate that the interaction between HVEM and BTLA is required for the optimal survival of effector CD8^+^ T cells during LM-OVA infection.

Interestingly, the numbers of memory CD8^+^ T cells found in infected mice that were treated with the 6A6 antibody were also lower. The treatment with the mAb one day before infection and again at day 2 p.i. (**Figure 7C**), or at days 4 and 6 p.i. (**Figure S4A**), led to reduced numbers of OVA-specific CD8^+^ T cells at day 70 p.i. Injection of the anti-BTLA antibody at days 8 and 10 after LM-OVA infection also led to reduced numbers of memory CD8^+^ T cells, further suggesting a role for HVEM-BTLA interaction during the contraction phase of the immune response (**Figure S4B**). HVEM is highly expressed on memory differentiated CD8^+^ T cells and therefore its interaction with BTLA may also be important for maintenance of these cells. To address this possibility we transferred WT OT1 cells into congenic hosts that were then infected with LM-OVA and treated with the IgG control or the anti-BTLA mAb at days 27, 30 and 34 p.i. Interestingly, blockade of the HVEM-BTLA interaction at these late times after LM-OVA infection did not affect the maintenance of memory OT1 cells. Indeed, equivalent percentages and numbers of the OT1 T cells were found in the spleen and small intestine of mice treated with the IgG or 6A6 mAb (**Figure 7D**). Thus, the critical role for HVEM-BTLA interaction in regulating effector and memory CD8^+^ T cell survival apparently occurs at the early phases of the immune response.

Because expression of BTLA on CD8^+^ T cells is not required for the accumulation of these cells during LM-OVA infection, the reduced numbers of OVA-specific T cells observed after 6A6 mAb treatment were likely related to the blockade of HVEM signals triggered in “*trans*” by BTLA expressed on host cells. However, it remained possible that the reduced number of OVA-specific cells after 6A6 treatment also may have been caused by unanticipated agonistic effects of the anti-BTLA mAb on BTLA^+^ CD8^+^ T cells, which would have inhibited the response of these cells. To evaluate this possibility, we carried out mAb blocking experiments in mice transferred with WT or BTLA-deficient OT1 cells. Seven days p.i, the number of OVA-specific T cells in the spleen of transferred animals was measured. Similar to our observations with endogenous polyclonal CD8^+^ T cells, the anti-BTLA mAb led to a significant reduction in the percentage (**Figure 7E**) and number (**Figure 7F** **)** of transferred WT OT1 cells. Notably, the administration of the 6A6 antibody also led to an equivalent decrease in the percentage and number of transferred *Btla* ^−/−^ OT1 cells (**Figure 7E,F**). These results indicate that the 6A6 mAb works independently of BTLA expression by the OT1 cells, and therefore its inhibitory effect on the CD8 response is not due to agonistic engagement of BTLA on CD8^+^ T cells.

The simplest and most cogent model to explain our findings is that BTLA expressed by APC engages HVEM expressed by an activated CD8^+^ T lymphocyte to promote the survival of effector and memory precursor cells. Because HVEM can interact with several binding partners, a less direct model remained possible, however, in which HVEM expression by CD8^+^ T cells alters effector and memory cell accumulation predominantly via interactions with LIGHT and/or CD160. In this model, BTLA, which is known to bind only to HVEM, could affect the accumulation of activated CD8^+^ T cells indirectly, through binding HVEM on another cell type. To test these possibilities, we performed adoptive co-transfer experiments of WT (CD45.1^+^CD45.2^+^) and *Hvem* ^−/−^ (CD45.2^+^) OT1 cells into CD45.1^+^ WT or *Btla* ^−/−^ hosts that were then infected with LM-OVA. As shown earlier, co-transfer of WT and *Hvem* ^−/−^ OT1 cells into WT hosts resulted in a dramatic reduction in the percentages of the HVEM-deficient OT1 cells in the spleen of the infected mice (**Figure 7G**). In these hosts, the average percentage of WT OT1 cells was 32-fold higher than that of the *Hvem* ^−/−^ cells (**Figure 7H**). In *Btla* ^−/−^ hosts, consistent with our previous results, the percentage of WT OT1 cells was significantly reduced compare to that observed in WT recipients (**Figure 7G**). It was notable, however, that the selective advantage of the WT over the HVEM-deficient OT1 cells was substantially reduced in *Btla^−/−^* hosts, such that it was only about two times higher (**Figure 7H**). These findings suggest that the reduction in WT OT1 cell numbers observed in infected *Btla* ^−/−^ hosts was related primarily to the absence of HVEM-mediated signaling in these T cells.

## Discussion

Here, we report the surprising finding that during oral infection with intracellular bacteria, *Listeria monocytogenes*, the BTLA-HVEM pathway plays a critical role in the adaptive immune system by promoting the survival of antigen-specific CD8^+^ T cells. *Hvem* ^−/−^ and *Btla^−/−^* mice did not show a significant difference in bacterial clearance at early times after oral administration (data not shown), but BTLA- and HVEM-deficient mice infected with LM-OVA had significantly reduced numbers of primary effector and memory CD8^+^ T cells, and specific blockade of the BTLA-HVEM interaction with an antagonist anti-BTLA mAb during the early stages of the immune response led to markedly reduced numbers of OVA-specific CD8^+^ T in WT mice infected with LM-OVA. We further show that HVEM expression on CD8^+^ T lymphocytes and BTLA expression by a different cell type are required for the optimal survival of effector and memory T cells. Because *Hvem* ^−/−^ OT1 cells failed to accumulate maximally at the peak of the immune response, despite their relatively normal proliferation and expansion rates observed early following antigen-stimulation, our results indicate that a survival defect likely is responsible for the decreased CD8^+^ T cell accumulation.

BTLA is emerging as an increasingly important immune regulator. It can function as an Ig super family ligand for HVEM that triggers activation of the NF-κB pathway [16]. Interestingly, because the same cell can express BTLA and HVEM, *in vitro* and *in vivo* experiments indicate that a *cis* interaction, *i.e.*, between molecules intrinsically expressed in the same cell, also can occur [17]. BTLA-mediated activation of NF-κB is only initiated, however, with BTLA interacting with HVEM in the conventional *trans* configuration, whereas in *cis* the interaction BTLA is primarily inhibitory for HVEM activation [17]. A role for BTLA in promoting T cell survival has been reported in several experiments [13], [14], [15], [16], although the opposite was found in one study [19]. In each of these reports, by contrast with our findings, BTLA expression on the responding T lymphocytes was critical. The contexts of the earlier studies, however, were entirely different, as in each case chronic inflammation models, as opposed to an acute infection, were analyzed. Interestingly, in a GVHD model, expression of a truncated form of BTLA lacking the intracellular signaling domain was sufficient to prolong the survival of the chronically stimulated *Btla* ^−/−^ donor T cells, suggesting that BTLA promotes T cell survival acting principally as a ligand for HVEM [15]. Because the *cis* interaction does not activate NF-κB [17], it is likely that engagement of HVEM in *trans* may have a pro-survival effect. This is supported by our previous observation where the administration of a BTLA-Fc fusion protein to T cell cultures enhanced the survival of *Btla* ^−/−^ T cells undergoing proliferation after *in vitro* anti-CD3 mAb stimulation [16]. In this experimental setup, the critical survival signal for HVEM-expressing T cells was provided by BTLA expressed on T lymphocytes, presumably during T cell -T cell encounters [15].

In the context of acute infection, we found that expression of BTLA on CD8^+^ T cells was not necessary for the accumulation of antigen-specific CD8^+^ T cells. Instead, BTLA expression on the host cells of infected mice was critical for the accumulation of HVEM^+^ CD8^+^ T cells following LM-OVA challenge. Similar results have also been found following systemic viral infection (Flynn R. et.al. co-submitted manuscript).

Our results indicate that the critical contribution of the BTLA-HVEM interaction for the accumulation of CD8^+^ T cells takes place at the early stages of the immune response. In both *Btla* ^−/−^ and *Hvem* ^−/−^ mice, the endogenous, polyclonal CD8^+^ T cell response to LM-OVA was significantly decreased at day 7 p.i. Also, the number of WT OT1 cells transferred into BTLA-deficient hosts, and *Hvem* ^−/−^ OT1 cells transferred into WT hosts, were substantially decreased at the peak of the immune response compared to controls. Additionally, blockade of the BTLA-HVEM interaction with the anti-BTLA antibody administrated early during LM-OVA infection led to a significant reduction in the number of OVA-specific CD8^+^ T cells at days 7 and 70 p.i. Therefore, the BTLA-HVEM interaction likely promotes the survival of activated CD8^+^ T cells acting early after antigen stimulation, near the peak of the immune response during the clonal expansion and contraction of the antigen-specific T cells. Because HVEM is expressed on naïve and memory CD8^+^ T cells, it is possible that HVEM influences CD8^+^ T cell survival differently at each specific phase of the immune response. A role for the BTLA-HVEM interaction in decreasing the memory CD8^+^ T cell pool, for example during homeostatic expansion, has been reported [20]. This was attributed to inhibitory signaling by BTLA expressed by the responding T lymphocytes. In our experiments, however, the pro-survival effect of HVEM expression by the CD8^+^ T cells was clearly dominant over the inhibitory effects of BTLA expression by these cells. This difference may reflect differences in the experimental setting, which in our case was distinguished by oral immunization with an infectious agent.

Besides BTLA, HVEM can also bind to LTα, LIGHT and CD160, and different and/or overlapping functions for these ligands are possible. Interestingly, recent reports from Ertl and colleagues showed that the viral ligand HSV-1 gD, which competes for HVEM binding with BTLA [21], [22], can enhance the adaptive immune response to viral antigens [23], [24]. In these studies, the more potent responses elicited by vaccines containing gD were presumed to work by blocking inhibitory interactions mediated by HVEM-BTLA and/or HVEM-CD160. Another possible interpretation of these results that is consistent with our findings, however, is that the more robust T responses observed with vaccines containing gD were caused by enhancement of HVEM^+^ antigen-specific T cell survival. In that context, we note that gD can function as an unconventional ligand for HVEM, and similarly to BTLA, it can trigger activation of the NF-κB-RelA signaling pathway [16]. Thus, BTLA, gD and potentially CD160, can function as agonistic ligands for HVEM, providing a mechanism, independent of TNF superfamily ligands, to promote stimulatory cosignaling required for effective T cell survival. HVEM activation by LIGHT could also be involved in different contexts, however, in promoting the survival of activated T lymphocytes during microbial infections. Whereas in studies performed *in vitro*, the costimulatory function of LIGHT was required for optimal CD8^+^ T cell activation, [25], [26], [27], [28], [29], LIGHT expression in mice was not required to control *Listeria* or Influenza A infections [28], [29]. It is possible that during natural bacteria or viral infections in *Light* ^−/−^ mice, BTLA and/or CD160 may signal through HVEM to compensate for the absence of LIGHT. In a lung inflammation model, however, a recent report showed that the function of LIGHT in engaging HVEM was essential [30]. As in the inflammatory models mentioned above, LIGHT expression by T lymphocytes was most important, providing another example in which HVEM engagement leading to the enhanced survival of activated lymphocytes, followed from T cell -T cell encounters.

In summary, our study has revealed a previously unappreciated role for the BTLA-HVEM pathway in the accumulation and likely the survival of adaptive immune cells required for host defense against microbial infections in the mucosa. We clearly demonstrated that the function of the BTLA-HVEM pathway is not limited to inhibitory signaling in the responding T lymphocytes, as BTLA expressed in “*trans*” by cells other than T lymphocytes promoted the survival and accumulation of HVEM^+^CD8^+^ T cells during *Listeria* infection. As the BTLA-HVEM pathway is highly conserved between mice and humans, we consider it likely that these molecules also regulate human CD8^+^ T cell survival during microbial infections. Therefore, manipulation of the HVEM-BTLA pathway with agonists against HVEM, such as HSV1-gD or others, which perhaps would block inhibitory BTLA signaling simultaneously, may be beneficial for improved vaccine efficacy, especially for the mucosal routes of infection that are taken by so many pathogens.

## Materials and Methods

### Ethics Statement

This study was carried out in strict accordance with the recommendations in the Guide for the Care and Use of Laboratory Animals of the animal Welfare Act and the National Institutes of Health. All animal protocols were approved by the Institutional Animal Care and Use Committee (IACUC) of the La Jolla Institute for Allergy and Immunology, San Diego (OLAW Assurance # A3779-01).

#### Mice

C57BL/6 (WT) and C57BL/6-SJL CD45.1 congenic mice were purchased from the Jackson Laboratories and maintained in our facility. *Btla^−/−^* [3] and *Hvem* ^−/−^ [9] mice were generously provided by Dr. Kenneth Murphy (Washington University St. Louis) and Dr. Klaus Pfeffer (Institute of Medical Microbiology, Universität Düsseldorf, Germany), respectively. *Btla* ^−/−^ and *Hvem* ^−/−^ mice were cross-bred with OVA_257–264_ peptide-specific T cell receptor-transgenic (OT1) mice to generate *Btla* ^−/−^ OT1 and *Hvem* ^−/−^ OT1 mice. C57BL/6 OT1 mice, CD45.1 congenic OT1 mice, CD45.1^+^CD45.2^+^ OT1 mice, *Btla* ^−/−^ OT1 mice and *Hvem* ^−/−^ OT1 mice were maintained by breeding under specific pathogen-free conditions at the La Jolla Institute for Allergy & Immunology.

#### Cell isolation, cell sorting and adoptive transfer

Cell sorting was done by flow cytometry using a FACSAria (Becton Dickinson). For adoptive transfer experiments, CD8β**^+^**CD44^low^ OT-I cells were sorted as naïve CD8^+^ OT-I cells. After sorting, cells were washed and 5×10^4^ cells were intravenously injected into recipient mice. For co-transfer experiments, 5×10^4^ cells with the corresponding congenic marker were mixed at 1∶1 ratios and injected into recipient mice as indicated above.

#### Bacterial infection

Infections with wild type LM-OVA were done as described previously [31]. LM-OVA was cultured in brain heart infusion broth. After culture, bacteria were washed and resuspended in Hanks’ balanced salt solution (HBSS) prior to infection. For immunization, 1×10^9^ LM-OVA were administrated to mice by oral gavage. Bacterial injection stocks were plated to confirm the CFU.

#### Antibody blocking experiments

Purified hamster-anti-mouse BTLA mAb (clone 6A6) and hamster IgG1 isotype control were purchased from Bio-X-Cell, Inc. (West Lebanon, NH). For blocking experiments at early stages of the immune response, mice were injected intraperitoneally with 100 µg of the anti-BTLA mAb administrated twice, either 1 day before and 2 days after infection, at days 4 and 6 post infection, or at days 8 and 10 after LM-OVA infection. For blocking experiments at the memory phase of the immune response, 100 µg of the anti-BTLA antibody were administrated at 20, 27, 30 and 34 days p.i.

#### CFSE labeling

Sorted OT1 cells were resuspended at a concentration of 1×10^7^ cells/ml in PBS and CFSE was added to a final concentration of 5 µM (Molecular Probe, Eugene, OR). After 10 min of incubation at 37°C, labeling was quenched with ice-cold DMEM medium with 10% FCS. Cells were washed three times with PBS before adoptive transfer.

#### IEL preparation

Isolation of IEL from small intestines was performed as described previously [32]. In brief, small intestines were removed and separated from Peyer’s patches. They were cut longitudinally and then into 0.5-cm pieces. The pieces were shaken for 40 min in Mg^2+^-free, Ca^2+^-free HBSS supplemented with 1 mM dithiothreitol and 5% FBS. Cells were collected from the washes and passed over a discontinuous 40–70% Percoll (Pharmacia Biotech, Piscataway, NJ) gradient at 900 *g* for 20 min. IEL were then isolated from the Percoll-gradient interface and washed in PBS.

#### Immunofluorescence staining and flow cytometry

Cells were pre-incubated with α-CD16/CD32 Fc-receptor antibody (2.4G2) to block Fc-antibody binding. Then, cells were stained in cold PBS containing 0.5% FBS and 0.05% sodium azide with the relevant labeled antibodies and tetramers. The following antibodies were used: CD8α (clone 53.6.7), CD8β (clone 53-5.8), CD44 (clone IM7), CD45.1 (clone A20), CD45.2 (clone 104), IFNγ (clone XMG1. 2), BTLA (clone 8F7) and HVEM (clone LH1). All primary antibodies were directly conjugated to fluorophores (BD PharMingen, San Diego, CA, or eBioscience, San Diego, CA). Endogenous OVA-specific CD8^+^ T cells were detected with PE-labeled K^b^/OVA_257–264_ tetramers. For intracellular staining of IFNγ, splenocytes, MLN cells, or IEL were stimulated with OVA_257–264_ peptide (5 µg/ml) for 5 h in the presence of brefeldin A, at 37°C. After surface staining, intracellular cytokine staining for IFNγ was performed using a Cytofix/Cytoperm Kit (BD PharMingen) according to the manufacturer’s directions. All the stained cells were analyzed on a FACSCalibur or LSRII^®^ flow cytometers (Becton Dickinson, San Jose, CA) and FlowJo software (Three Star, Ashland, OR).

#### Statistical analysis

Differences between groups were evaluated for statistical significance by the nonparametric Mann-Whitney test or two-tailed unpaired Student’s *t* test. Results are expressed as mean ± SEM. p-values <0.05 were considered significant.

## Supporting Information

Figure S1 **BTLA and HVEM expression in naïve and memory CD8^+^ T cells.** Total splenocytes isolated from naïve WT mice were stained with antibodies against BTLA and HVEM and the expression of the two proteins was monitored by flow cytometry. BTLA and HVEM were both detected at the surface of CD8^+^ T cells, although CD44^Hi^ cells expressed slightly higher levels of BTLA than naïve CD44^low^ cells. Conversely, the expression of HVEM in the CD44^Hi^ subset was lower than that observed in naïve CD8^+^ T lymphocytes. Representative data are shown.
(EPS)

Figure S2 **IFNγ** **production by CD8^+^ T cells isolated from the MLN of WT and** ***Btla*** **^−/−^ animals, 7 days post LM-OVA infection.** WT or *Btla* ^−/−^ mice were orally infected with LM-OVA. Seven days post-infection (p.i.), MLN cells were isolated and re-stimulated *ex vivo* with the SIINFEKL peptide epitope, in the presence of brefeldin A. Following re-stimulation, the percentage and absolute number of WT and *Btla* ^−/−^ CD8^+^ T cells secreting IFNγ was calculated by intracellular IFNγ staining. Data pooled from three independent experiments are shown.
(EPS)

Figure S3 **Expression of effector CD8 T cell differentiation markers on WT and** ***Hvem*** **^−/−^ OT1 cells.** The expression of cell surface markers KLRG1, CD127, CD62L, CD43 and CD122, on the surface of WT and *Hvem* ^−/−^ OT1 cells was analyzed at days 5, 7, 9, 12 and 15 after LM-OVA infection. Data show the percentages of CD45.2^+^ OT1 cells (open histograms) expressing the markers indicated above. Filled histograms correspond to expression levels in endogenous CD45.1^+^ CD8 cells from the host mice. Representative data is shown.
(EPS)

Figure S4 **Reduced percentage of memory OT1 cells in WT recipient mice treated with the anti-BTLA mAb.** To determine if the critical contribution of the BTLA-HVEM interaction take place before or after the peak of the immune response, WT mice transferred with OT1 cells were treated with the 6A6 anti-BTLA mAb at days 4 and 6 post infection (A), or at days 8 and 10 post infection (B), and the percentage of memory OT1 cells was measured at day 70 p.i. Data shown correspond to the average of 4 mice per group.
(EPS)
